# Correction: Prevalence of prediabetes and type 2 diabetes mellitus in south and southeast Asian women with history of gestational diabetes mellitus: Systematic review and meta-analysis

**DOI:** 10.1371/journal.pone.0304170

**Published:** 2024-05-16

**Authors:** Chockalingam Shivashri, Wesley Hannah, Mohan Deepa, Yonas Ghebremichael-Weldeselassie, Ranjit Mohan Anjana, Ram Uma, Viswanathan Mohan, Ponnusamy Saravanan

The Data Availability statement for this paper is incorrect. The correct statement is: The dataset pertaining to our systematic review is available in the Open Science Framework repository: https://doi.org/10.17605/OSF.IO/DV5PU.
