# Supplementary figures and images for: Prevalence of prediabetes and type 2 diabetes mellitus in south and southeast Asian women with history of gestational diabetes mellitus: Systematic review and meta-analysis
Source: PLoS One. 2022 Dec 12;17(12):e0278919. doi: 10.1371/journal.pone.0278919 (PMC9744276; doi:10.1371/journal.pone.0278919)

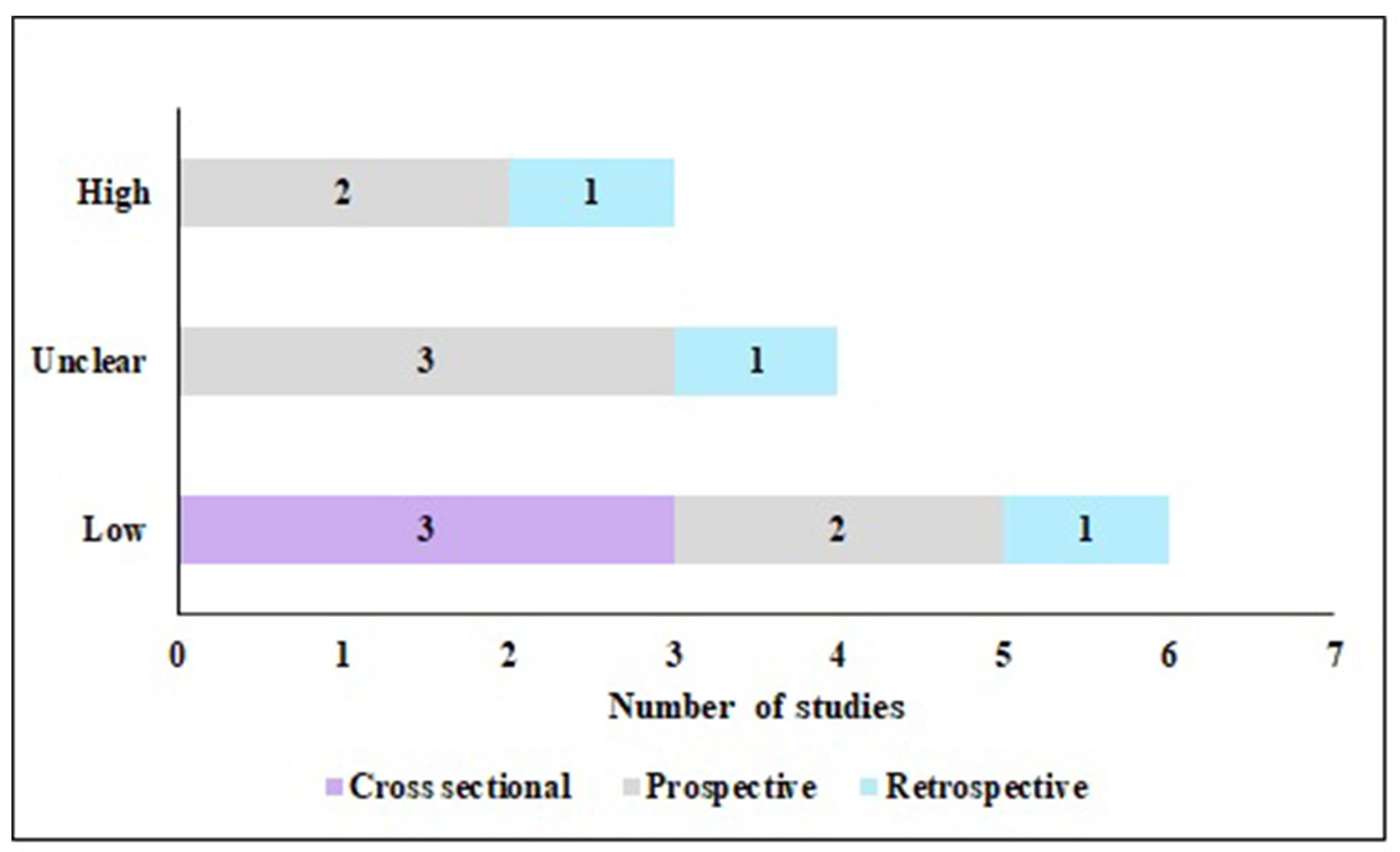

Supplement: S1 Fig — (TIF) [file pone.0278919.s001.tif]

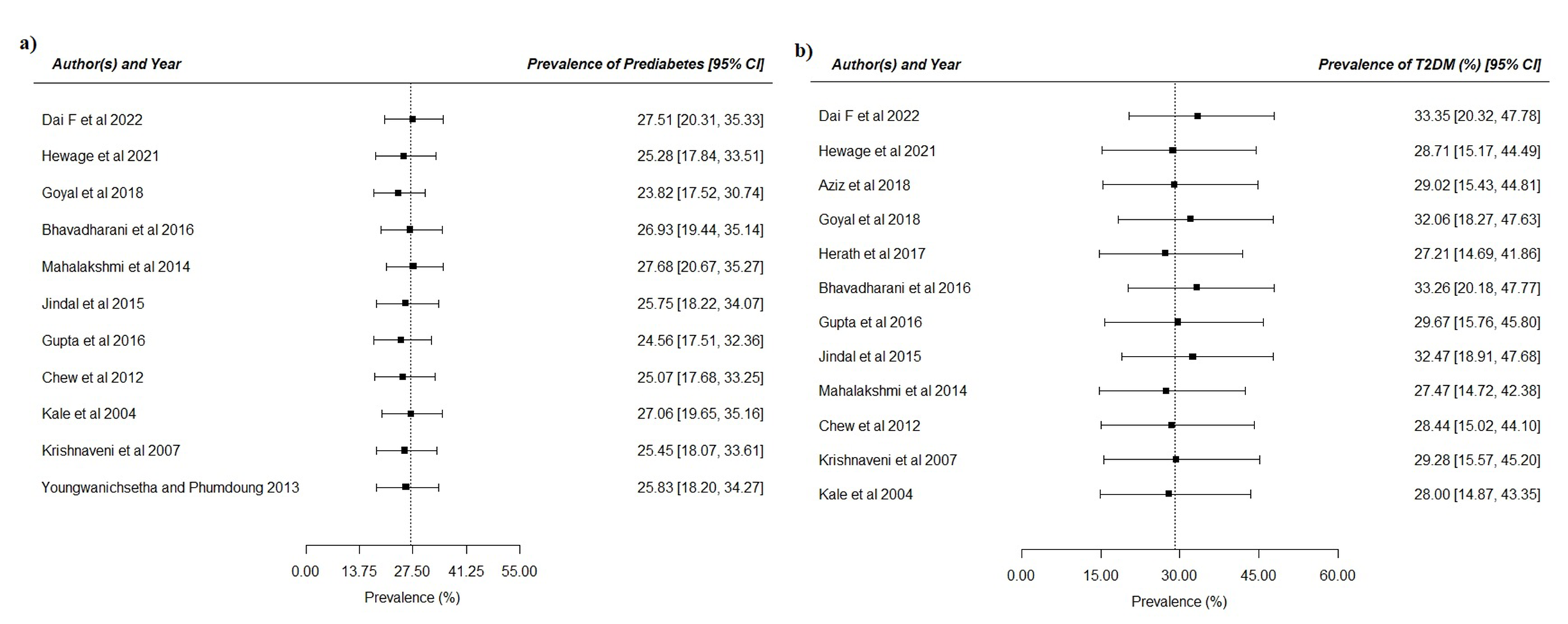

Supplement: S2 Fig — Sensitivity analyses for the prevalence of (a) prediabetes and (b) T2DM. (TIF) [file pone.0278919.s002.tif]

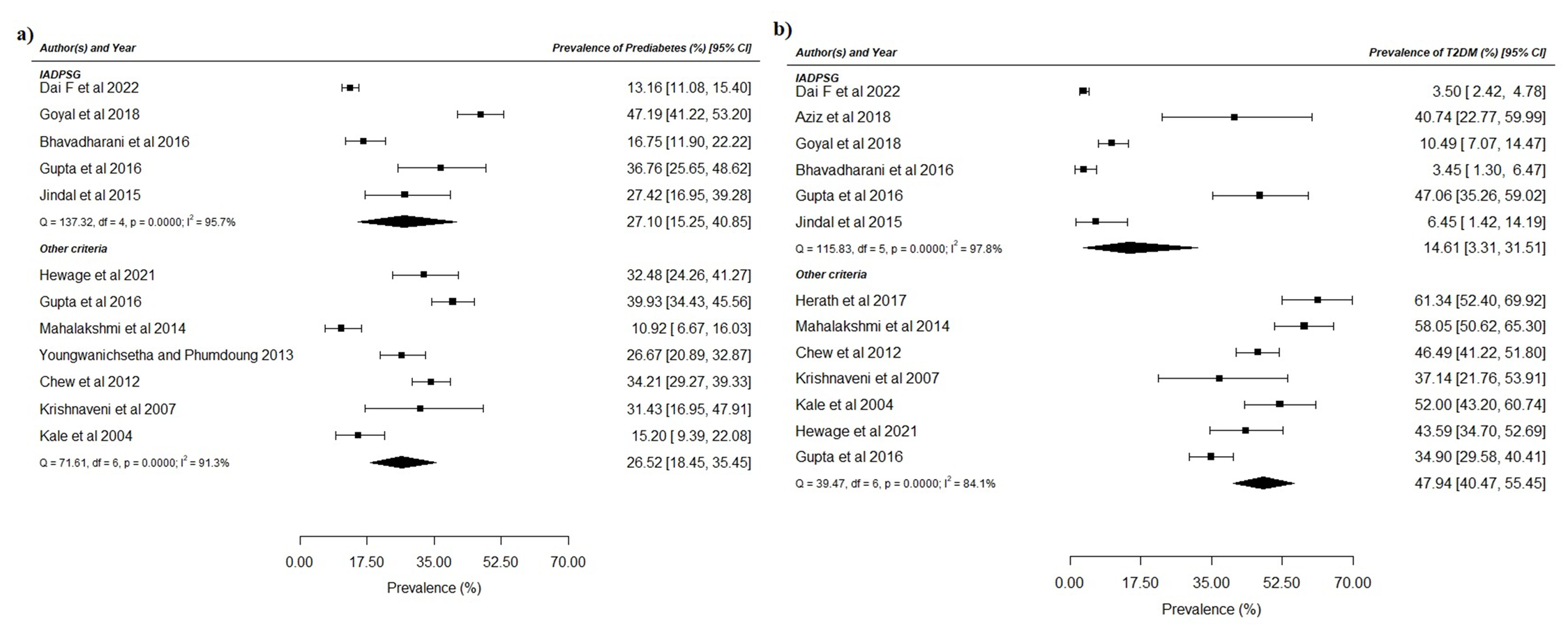

Supplement: S3 Fig — Prevalence of (a) prediabetes and (b) T2DM based on the diagnostic criteria of GDM. (TIF) [file pone.0278919.s003.tif]

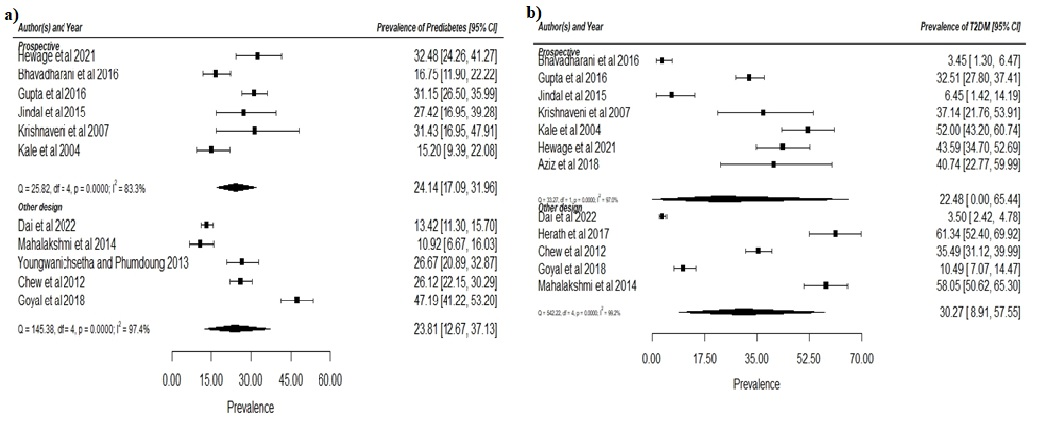

Supplement: S4 Fig — Prevalence of (a) design prediabetes and (b) T2DM based on the study. (TIF) [file pone.0278919.s004.tif]

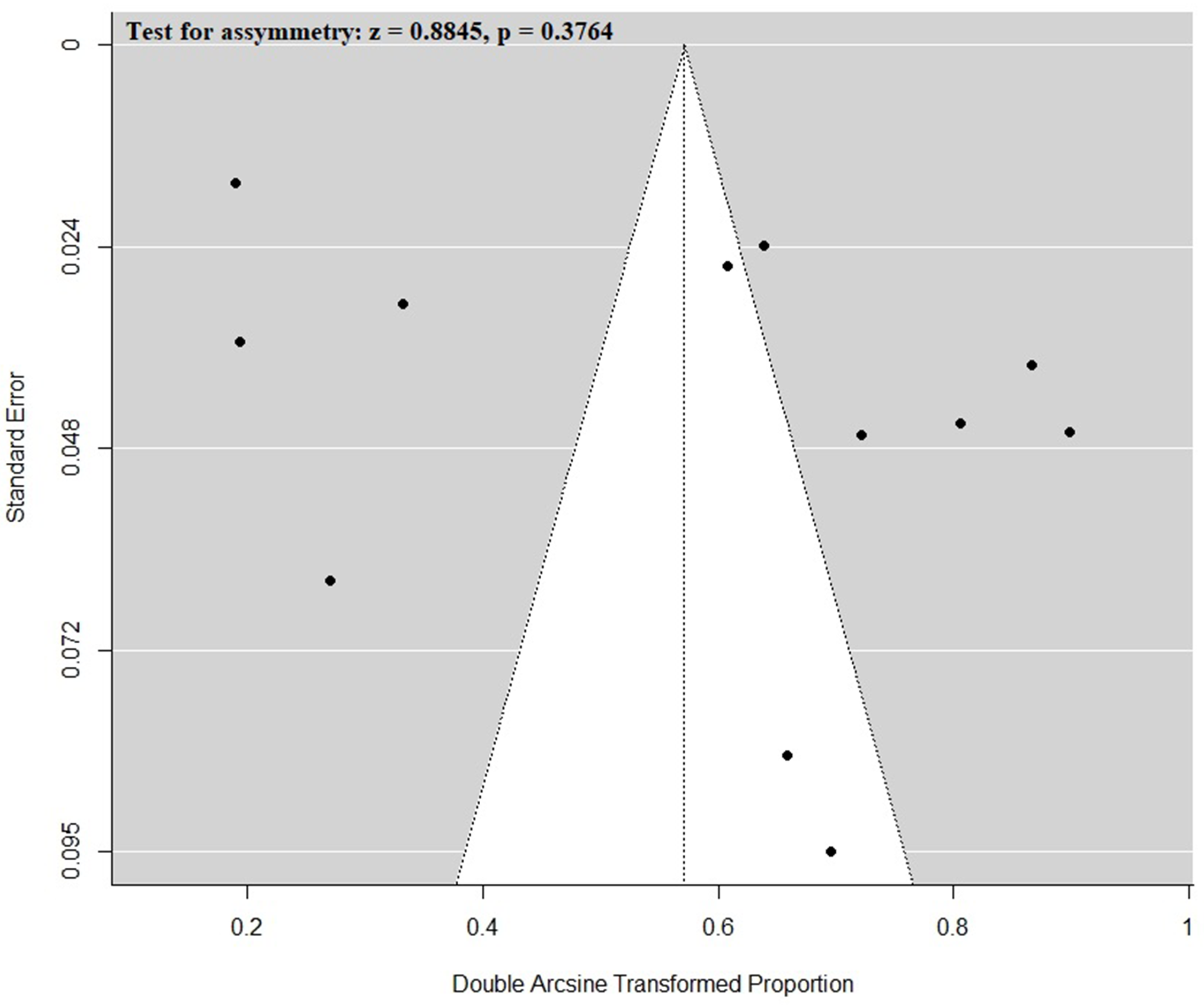

Supplement: S5 Fig — (TIF) [file pone.0278919.s005.tif]
