# Supplementary material for: Prevalence of prediabetes and type 2 diabetes mellitus in south and southeast Asian women with history of gestational diabetes mellitus: Systematic review and meta-analysis
Source: PLoS One. 2022 Dec 12;17(12):e0278919. doi: 10.1371/journal.pone.0278919 (PMC9744276; doi:10.1371/journal.pone.0278919)
Supplement: S1 Table — (DOCX) [file pone.0278919.s006.docx]

|  | **Selection** | | | | **Comparability** | **Outcome** | | | **Total** | **Risk of bias** |
| --- | --- | --- | --- | --- | --- | --- | --- | --- | --- | --- |
|  | **Representativeness of the exposed cohort** | **Selection  of non exposed cohort** | **Ascertainment  of exposure** | **Demonstration  that outcome of interest was not present at the start of the study** | **Comparability  of cohorts on the basis of the design or analysis** | **Assessment of outcome** | **Was follow-up long enough for outcomes to occur** | **Adequacy of follow up cohorts** |  |  |
| Dai et al 2022[33] |  |  | ***** | ***** |  | ***** |  |  | 3 | high |
| Hewage et al 2021[34] |  |  | ***** | ***** |  | ***** | ***** | ***** | 5 | unclear |
| Aziz et al 2018[35] |  |  | * | * |  | * |  |  | 3 | high |
| Herath et al 2017[37] | * | * | * | * | * | * | * | * | 8 | low |
| Gupta et al 2017[38] | * |  | * | * |  | * | * |  | 5 | unclear |
| Bhavadharani et al 2016[26] | * |  | * | * |  | * |  | * | 5 | unclear |
| Jindal et al 2015[39] |  |  | * | * |  | * |  |  | 3 | high |
| Mahalakshmi et al 2014[40] |  |  | * | * |  | * | * | * | 5 | unclear |
| Krishnaveni et al 2007[43] | * | * | * | * | ** | * | * | * | 9 | low |
| Kale et al 2004[44] | * | * | * | * | * | * | * | * | 8 | low |

**S1 Table. Quality assessment of cohort studies using Newcastle Ottawa scale (n=10).**
