# Supplementary material for: Prevalence of prediabetes and type 2 diabetes mellitus in south and southeast Asian women with history of gestational diabetes mellitus: Systematic review and meta-analysis
Source: PLoS One. 2022 Dec 12;17(12):e0278919. doi: 10.1371/journal.pone.0278919 (PMC9744276; doi:10.1371/journal.pone.0278919)
Supplement: S2 Table — (DOCX) [file pone.0278919.s007.docx]

**S2 Table. Quality assessment of cross-sectional studies (n=3)**

|  | **Risk of bias item** | **Goyal et al 2018[36]** | **Chew et al 2012[42]** | **Youngwanichsetha and Phumdoung 2013[41]** |
| --- | --- | --- | --- | --- |
| 1 | Was the study’s target population a close representation of the national population in relation to relevant variables, e.g. age, sex, occupation | Yes | Yes | Yes |
| 2 | Was the sampling frame a true or close representation of the target population? | No | Yes | Yes |
| 3 | Was some form of random selection used to select the sample, OR, was a census undertaken? | No | Yes | Yes |
| 4 | Was the likelihood of non-response bias minimal? | Yes | No | Yes |
| 5 | Were data collected directly from the subjects (as opposed to a proxy)? | Yes | Yes | Yes |
| 6 | Was an acceptable case definition used in the study? | Yes | Yes | Yes |
| 7 | Was the study instrument that measured the parameter of interest (e.g. prevalence of low back pain) shown to have reliability and validity (if necessary)? | Yes | Yes | Yes |
| 8 | Was the same mode of data collection used for all subjects? | Yes | Yes | Yes |
| 9 | Was the length of the shortest prevalence period for the parameter of interest appropriate? | Yes | Yes | No |
| 10 | Were the numerator(s) and denominator(s) for the parameter of interest appropriate? | Yes | Yes | Yes |
|  | Risk of bias | low | low | low |
