# Supplementary material for: Prevalence of prediabetes and type 2 diabetes mellitus in south and southeast Asian women with history of gestational diabetes mellitus: Systematic review and meta-analysis
Source: PLoS One. 2022 Dec 12;17(12):e0278919. doi: 10.1371/journal.pone.0278919 (PMC9744276; doi:10.1371/journal.pone.0278919)
Supplement: S3 Table — (DOCX) [file pone.0278919.s008.docx]

| **Study** | **N** | **T2DM conversion in the GDM group** | **T2DM in the Non-GDM group** | **Calculated relative risk** | **Confounding variables** |
| --- | --- | --- | --- | --- | --- |
| Herath et al 2017[37] | 575 | 73/119 | 18/456 | 10.52 [6.20, 17.83] | Age at delivery, treatment with Insulin during index  Pregnancy, Birth weight, History of GDM in previous pregnancies, Diabetes in a first degree relative at index  Pregnancy, Parity of index pregnancy, Gestational age at delivery |
| Krishnaveni et al  2007[43] | 524 | 13/35 | 8/489 | 22.70 [10.09, 51.10] | NA |
| Kale et al 2004[44] | 365 | 65/125 | 14/240 | 13.17 [8.13, 21.35] | NA |

**S3 Table. Relative risk of T2DM in women with history of GDM compared with healthy controls in SA and SEA**

******NA -Not applicable*
