# Supplementary material for: Prevalence of prediabetes and type 2 diabetes mellitus in south and southeast Asian women with history of gestational diabetes mellitus: Systematic review and meta-analysis
Source: PLoS One. 2022 Dec 12;17(12):e0278919. doi: 10.1371/journal.pone.0278919 (PMC9744276; doi:10.1371/journal.pone.0278919)
Supplement: S1 Appendix — (DOCX) [file pone.0278919.s009.docx]

**S1 Appendix. Detailed search strategy**

**For Medline and EMBASE**

((gestational diabetes.mp. or exp Diabetes, Gestational/) AND ((postpartum.mp. or exp Postpartum Period/ postnatal.mp.) OR (exp Postnatal Care/) OR (post-natal.mp.)) AND (type 2 diabetes.mp. or exp Diabetes Mellitus, Type 2/) AND (exp Asia, Western/ or exp Asia, Southeastern/))

**For CINHAL**

(((gestational diabetes mellitus" OR (MH "Diabetes Mellitus, Gestational"))AND ((MH "Postnatal Period+") OR(MH "Postnatal Care+") OR "postpartum or postnatal or after pregnancy or after birth")) AND (("Diabetes Mellitus, Type 2")OR ("type 2 diabetes mellitus")) AND ((MH” Asia, Western+") OR (MH"Asia, Southeastern+")))

**For Web of knowledge**

("gestational diabetes mellitus" or "gdm" or "gestational diabetes") AND TOPIC: (postpartum or postnatal or post-natal or post-partum) AND TOPIC: ("type 2 diabetes" or "t2dm" or "type 2 diabetes mellitus") AND TOPIC: (Afghanistan or Pakistan or Bangladesh or Bhutan or Maldives or Nepal or India or Vietnam or Thailand or Indonesia or Philippines or Singapore or Malaysia)

Journal articles, English only
